# Mercury and Selenium in Stranded Indo-Pacific Humpback Dolphins and Implications for Their Trophic Transfer in Food Chains

**DOI:** 10.1371/journal.pone.0110336

**Published:** 2014-10-13

**Authors:** Duan Gui, Ri-Qing Yu, Yong Sun, Laiguo Chen, Qin Tu, Hui Mo, Yuping Wu

**Affiliations:** 1 Guangdong Provincial Key Laboratory of Marine Resources and Coastal Engineering, School of Marine Sciences, Sun Yat-Sen University, Guangzhou, China; 2 Department of Biology, University of Texas at Tyler, Tyler, Texas, United States of America; 3 Urban Environment and Ecology Research Center, South China Institute of Environmental Sciences (SCIES), Ministry of Environmental Protection, Guangzhou, China; 4 South China Botanical Garden, Chinese Academy of Sciences, Guangzhou, China; Oak Ridge National Laboratory, United States of America

## Abstract

As top predators in the Pearl River Estuary (PRE) of China, Indo-Pacific humpback dolphins (*Sousa chinensis*) are bioindicators for examining regional trends of environmental contaminants in the PRE. We examined samples from stranded *S. chinensis* in the PRE, collected since 2004, to study the distribution and fate of total mercury (THg), methylmercury (MeHg) and selenium (Se) in the major tissues, in individuals at different ages and their prey fishes from the PRE. This study also investigated the potential protective effects of Se against the toxicities of accumulated THg. Dolphin livers contained the highest concentrations of THg (32.34±58.98 µg g^−1^ dw) and Se (15.16±3.66 µg g^−1^ dw), which were significantly different from those found in kidneys and muscles, whereas the highest residue of MeHg (1.02±1.11 µg g^−1^ dw) was found in dolphin muscles. Concentrations of both THg and MeHg in the liver, kidney and muscle of dolphins showed a significantly positive correlation with age. The biomagnification factors (BMFs) of inorganic mercury (Hg_inorg_) in dolphin livers (350×) and MeHg in muscles (18.7×) through the prey fishes were the highest among all three dolphin tissues, whereas the BMFs of Se were much lower in all dolphin tissues. The lower proportion of MeHg in THg and higher Se/THg ratios in tissues were demonstrated. Our studies suggested that *S. chinensis* might have the potential to detoxify Hg via the demethylation of MeHg and the formation of tiemannite (HgSe) in the liver and kidney. The lower threshold of hepatic THg concentrations for the equimolar accumulation of Se and Hg in *S. chinensis* suggests that this species has a greater sensitivity to THg concentrations than is found in striped dolphins and Dall’s porpoises.

## Introduction

Mercury (Hg) in its inorganic form is a ubiquitous pollutant that is globally distributed by atmospheric transportation. Less toxic Hg(II) in environments can easily be converted into the highly toxic methylmercury (MeHg), primarily by sulfate- and iron-reducing bacteria and methanogens [1]–[7] through the putative Hg methylation genes *hgc*A and *hgc*B via the acetyl CoA pathway [5]. MeHg is a strong neurotoxic substance; once it is bioavailable in aquatic ecosystems, it can be bioaccumulated and biomagnified quickly through aquatic food webs, which creates a health threat to humans and aquatic mammals such as dolphins [3].

The estuary of the Pearl River, which is the third longest river in China, is a traditional nursery for fisheries and provides an ideal habitat for Indo-Pacific humpback dolphins (*Sousa chinensis*). The Pearl River Estuary (PRE) region contains a group of cities that include Guangzhou, Shenzhen and Hong Kong, forming one of the largest local and global economic hubs in southern China. Rapid industrial development and urbanization in recent decades have undermined the habitats of local fish and dolphins in the estuary. *S. chinensis* is considered one of the most endangered species (the National Key Species for Protection, Grade 1) in China. The Chinese government has delimitated the PRE as a national nature reserve for *S. chinensis*. Recent studies have indicated a decreasing trend of total mercury (THg) concentration in sediments with distance away from the PRE and toward the South China Sea [8] and showed an accelerated input of THg in sediment cores in recent decades [9]. These results suggest that THg contamination in this region has been strongly correlated with industrial development. Contamination by Hg(II) and MeHg was also observed in surface water in the tributary (e.g., Dong River) of the Pearl River Delta. However, THg and MeHg contamination in Indo-Pacific humpback dolphins and the interaction between THg toxicity and Se accumulation in their bodies have not been systematically studied in this ecosystem [10], [11].

In aquatic ecosystems, dolphins are top predators that have long lifespans, which increases their potential to accumulate Hg(II) and MeHg [12]. MeHg is easily accumulated in the livers of cetaceans, likely as a result of the ability of the liver to store and biotransform toxic contaminants [13]. The accumulation of Hg(II) and MeHg might cause detrimental effects on the reproduction system, immune responses, central nervous system and organs such as the liver and kidneys [14], [15].

Previous studies have reported that selenium (Se) can mitigate the toxicity of MeHg in the liver of cetaceans by forming a highly insoluble Se–Hg compound after demethylating MeHg [16], [17]. The presence of the nontoxic Se–Hg compound was confirmed in the cytoplasm of hepatic cells in *Stenella coeruleoalba,* and an equimolar ratio between Se and THg was reported in liver tissue with a high THg concentration [18], [19]. This phenomenon likely explains the ability of cetaceans to tolerate high THg concentrations without directly observable adverse effects and exhibit a low MeHg residue in the brain tissue. Se is a micronutrient that plays an important role in enzymes (e.g., glutathione peroxidase) in the maintenance of normal organ functions. Adverse biological effects occur when there is a deficiency of bioavailable Se, and an Hg-induced Se deficiency can lead to MeHg or THg toxicity [20]. The present investigation examined samples from dolphins stranded in the PRE, which have been collected since 2004, to study the distribution and fate of toxic pollutants (THg, MeHg and Se) in the major organs, in individuals at different ages and in their prey fishes from the PRE. The aims of the study were also to investigate the potential protective effects of Se against the toxicities of accumulated THg, and to provide evidence for the conservation of this endangered species.

## Materials and Methods

### Ethics Statement

This study on the Indo-Pacific humpback dolphins was approved by the Ministry of Agriculture of Chinese government under permit number 2003-54. The protocol was specifically verified by the Administration of Ocean and Fisheries of Guangdong Province, China under permit number 1999-583. No issue on ethics was concerned in this study.

### Samples and chemical analysis

**Figure 1:**
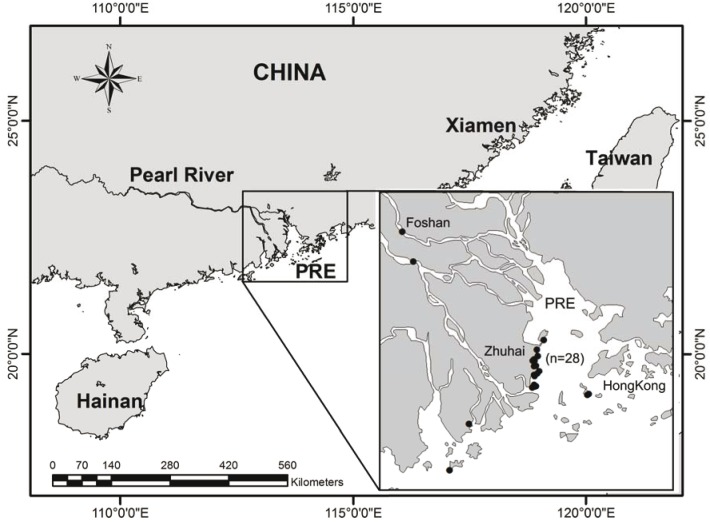
Sampling sites in the Pearl River Estuary (PRE) where the stranded Indo-Pacific humpback dolphins (n = 28) were collected from 2004 to 2012.

The tissue samples of *S. chinensis* were collected from dead and stranded animals along the PRE of the South China Sea from 2004 to 2012 (**Fig. 1**). Before performing the necropsies, the biological parameters were measured and tooth specimens were acquired to determine the age of specific individuals according to the method described by Jefferson [21] and Myrck et al. [22]. According to the report from Jefferson et al. [23], male dolphins inhabiting the PRE reach sexual maturity at 12–14 years, whereas females generally reach maturity at 9–10 years. Accordingly, the specimen assigned ID numbers from 21 to 28 in the present study were considered adults, and those with assigned numbers 1 to 20 were considered juveniles (**Table S1**). The liver, kidney and muscle tissue samples were placed in clean and acid-washed plastic bags and stored at −20°C immediately after collection. Information based on previous studies [21], [24] indicated that the Indo-Pacific humpback dolphins have specific preferences for prey fishes. Based on that, 13 species of fish were collected from the PRE. Whole-body fish samples were smashed for metal analysis. All samples (dolphin and fish) were kept frozen until processing.

Samples were processed and prepared by a method similar to that of previous studies [25], in which portions of the different tissue samples were freeze-dried (Freeze-drying system, Labconco, Kansas City, Missouri, USA) for 48 h at 40–133×10^−3^ mBar and −49°C. The dried samples were then ground with an automatic agate mortar (Retsch, Germany) for 10 min. The concentration of THg in the dried tissue samples was measured without pretreatment or digestion by using the Hydra-C Automated Direct Hg Analyzer (Teledyne Instruments, Leeman Labs, USA). All specimens were analyzed in batches that included a procedural blank and standard reference material DORM-3 (National Research Council of Canada, Canada). The procedural blank and the reference material were treated and measured in the same way as the tissue samples. To analyze the MeHg concentration, tissue samples were digested in a KOH–methanol solution at 65°C for 4 h. The extractants were subjected to aqueous ethylation, separation by gas chromatography and detection by cold vapor atomic fluorescence spectrometry (CVAFS) (TEKRAN Model 2700 with an automated MERX purge and trap system, Brooks Rand Labs, USA) modified from USEPA method 1630 [26].

The digestion and preparation of dried tissue samples for Se analysis followed the methods described by Hung et al. [10]. Approximately 0.2 g of tissue samples was weighed in Teflon digestion tubes and soaked overnight in a mixture of 2 ml of double-distilled deionized water (3-D water) and 5 ml of 70% nitric acid (Merck, Germany). The digestion tubes were then sealed, placed in a microwave oven (Xin Tuo, model XT-9912, China) and subjected to a pressure increase of 65 psi for 15 m. After cooling, 2 ml H_2_O_2_ was added to the sample solution. The pressure was then increased to 65 psi for 15 m. The resulting digests were cooled and filtered through disposable syringe filter discs (0.45 µm pores, 25 mm diameter, with a mixed cellulose-ester filtering material, Jing Teng, China) equipped with 50-ml plastic syringes. The filtrates were transferred to 25-ml volumetric flasks and diluted with 3-D water. The samples were kept in acid-washed PVC tubes at 4°C prior to trace element analysis. The concentration of Se was measured by an inductively coupled plasma mass spectrometer (ICP-MS) (Agilent 7700, USA) with the procedural blank and reference TORT-2 included with every batch of tissue samples.

### Quality assurance/quality control (QA/QC)

The precision and accuracy of the analytical methods were determined and monitored using the certified material TORT-2 (lobster hepatopancreas) and DORM-3 (fish protein) from the National Research Council of Canada. The recovery rates for THg, MeHg and Se were approximately 95.3%, 94.9% and 94.3%, respectively.

### Statistical analysis

Statistical analyses were performed using SPSS (Statistical Package for the Social Sciences) software (StatSoft, ver. 22, USA), and the level of statistical significance was defined as *p*<0.05. Grubbs’ test was used to identify outliers, which were removed before further calculations. The Kolmogorov–Smirnoff test was used to assess the normality of the data distribution; if the data were not in normality, all the data were log_10_-transformed to improve normality prior to analysis to best fit the underlying assumptions of the analysis of variance. The correlation between measured parameters was assessed by coefficient of determination (R^2^). Differences in the concentrations of MeHg, THg and Se were analyzed among the groups (adult males, adult females, juvenile males and juvenile females) by using one-way ANOVA followed by Tukey’s post-hoc tests. The contaminant concentrations of the stranded dolphins that exhibited a large dispersion were analyzed by non-parametric tests.

## Results

The total Hg, Se and MeHg concentrations and their related ratios were determined in the tissue samples from the liver (n = 28), kidney (n = 22) and muscle (n = 15) of Indo-Pacific humpback dolphins (**Table 1**). The mean concentrations of THg, Se and MeHg in 13 fish species from the PRE were summarized in **Table 2**. The average THg concentration in the prey fish species was 0.146 µg g^−1^ dw, with a range from 0.062 to 0.303 µg g^−1^ dw, and the average Se concentration was 2.23 µg g^−1^ dw, with a range from 1.93 to 3.34 µg g^−1^ dw. No significant differences in concentrations were found for the different fish species except the predatory species *Arius sinensis* and *Pampus argenteus*, which showed high THg concentrations. The potential biomagnification factor of inorganic mercury (Hg_inorg_) in the dolphin tissue from the prey fishes was the highest in the liver tissue (350-fold) and the lowest in the muscle tissue (4.78-fold) (**Table 2**). For MeHg, dolphin muscle tissue had the highest biomagnification factor (18.7-fold), followed by liver tissue (14.3-fold) and kidney tissue (9.6-fold). Overall, Se in dolphin tissues showed the lowest potential for biomagnification of the three contaminants. For all dolphin individuals, muscle represents around 30% of total body mass, while other tissues (e. g., liver and kidney) contribute much less to the total body weight (<5%).

**Table 1:** The mean concentrations and standard deviations (SD), in µg g^−1^ dry weight, of total mercury (THg), selenium (Se) and methyl mercury (MeHg); the molar ratio (%) of Se to THg; and the percentage (%) of MeHg/THg in liver, kidney and muscle tissue of Indo-Pacific humpback dolphins from the Pearl River Estuary (PRE), China.

		Liver (n = 28)					Kidney (n = 22)					Muscle (n = 15)				
		THg	Se	MeHg	Se:THg	MeHg:THg	THg	Se	MeHg	Se:THg	MeHg:THg	THg	Se	MeHg	Se:THg	MeHg:THg
JM (n = 13)	Mean	4.24	5.63	0.49	8.51	25	1.27	5.67	0.26	37.7	43.4	0.58	1.52	0.48	10.6	78.7
	SD	5.12	3.54	0.35	5.90	14.5	1.65	2.86	0.21	27.8	22.2	0.40	0.42	0.36	6.67	12.9
JF (n = 7)	Mean	2.27	5.09	0.47	7.14	23.4	1.73	6.95	0.36	15.46	27.5	1.29	2.78	0.43	7.82	66.9
	SD	1.36	1.20	0.15	2.59	6.66	1.14	1.83	0.16	8.6	11.9	1.91	2.67	0.34	2.64	32.3
	*p* values	n.s.	n.s.	n.s.	n.s.	n.s.	n.s.	n.s.	n.s.	n.s.	n.s.	n.s.	n.s.	n.s.	n.s.	n.s.
AM (n = 3)	Mean	84.3	35.6	1.56	1.07	1.88	8.21	10.3	0.98	3.13	11	2.27	1.61	2.03	0.90	89.4
	SD	8.19	6.93	0.44	0.14	0.64	1.19	2.68	0.35	0.35	3.39	0	0	0	0	0
AF (n = 5)	Mean	116	41.8	1.56	0.99	1.89	13.4	11.1	1.06	2.29	7.42	3.18	1.43	2.72	0.79	85.6
	SD	81.8	24.9	0.46	0.11	0.91	5.16	2.45	0.60	0.47	2.18	1.05	0.24	0.94	0.21	6.47
	*p* values	n.s.	n.s.	n.s.	n.s.	n.s.	n.s.	n.s.	n.s.	n.s.	n.s.	n.s.	n.s.	n.s.	n.s.	n.s.
M (n = 16)	Mean	3.19	5.63	0.60	9.08	20.7	2.87	6.73	0.43	29.7	36.1	0.82	1.53	0.70	9.01	80.4
	SD	3.76	3.54	0.44	5.76	15.9	3.32	3.42	0.39	28.4	23.7	0.70	0.39	0.64	7.07	12.5
F (n = 12)	Mean	31.6	20.4	0.93	4.92	14.4	6.90	8.79	0.67	9.61	18.6	2	2.2	1.29	4.81	73.9
	SD	50.3	24.2	0.62	3.90	11.8	6.77	2.96	0.54	9.17	13.4	1.88	2.13	1.28	4.01	27.4
	*p* values	n.s.	0.016	n.s.	n.s.	n.s.	n.s.	n.s.	n.s.	n.s.	n.s.	n.s.	n.s.	n.s.	n.s.	n.s.
All (n = 28)	Mean	32.3	15.2	0.79	6.03	18.5	4.52	7.57	0.53	21.5	29.0	1.45	1.89	1.02	6.75	76.9
	SD	59.0	19.4	0.61	5.40	15.3	5.53	3.47	0.49	25.3	22.5	1.62	1.69	1.11	6.26	23.3

JM: juvenile male (<12 years); JF: juvenile female (<9 years); AM: adult male (>12 years); AF: adult female (>9 years).

n.s.: not significant.

**Table 2:** The mean concentrations and standard deviations (SD), in µg g^−1^ dry weight, of total mercury (THg), inorganic mercury (Hg_inorg_), methylmercury (MeHg), selenium (Se), and the percentage (%) of MeHg/THg in the prey fishes (whole body) for Indo-Pacific humpback dolphins and their average biomagnification factors (BMFs) in the dolphin tissues collected from the Pearl River Estuary (PRE), China.

Species		Family	Samplenumber	THg		Hg_inorg_		MeHg		Se		MeHg/THg
				Mean	SD	Mean	SD	Mean	SD	Mean	SD	Mean
*Johnius belengerii*	Sciaenidae		3	0.146	0.075	0.100	0.079	0.046	0.01	2.51	0.41	25
*Collichthys lucidus*	Sciaenidae		4	0.134	0.106	0.073	0.082	0.060	0.026	2.61	0.47	45
*Clupanodon thrissa*	Clupeidae		2	0.182	0.129	0.147	0.127	0.036	0.002	1.87	0.58	20
*Coilia mystus*	Engraulidae		5	0.158	0.152	0.121	0.144	0.037	0.008	1.99	0.43	23
*Pampus argenteus*	Stromateidae		3	0.221	0.111	0.189	0.119	0.032	0.008	2.22	0.22	15
*Harpadon nehereus*	Synodontidae		2	0.079	0.024	0.030	0.016	0.048	0.009	2.06	0.63	61
*Cynoglossus bilineatus*	Cynoglossidae		3	0.077	0.024	0.039	0.015	0.038	0.015	2.21	0.33	50
*Sillago sihama*	Sillaginidae		3	0.166	0.036	0.029	0.017	0.137	0.044	1.99	0.27	83
*Arius sinensis*	Ariidae		2	0.303	0.007	0.247	0.006	0.056	0.001	1.93	0.08	19
*Selaroides leptolepis*	Carangidae		2	0.118	0.065	0.079	0.058	0.039	0.007	1.99	0.40	33
*Mugil cephalus*	Mugilidae		2	0.062	0.002	0.033	0.005	0.030	0.003	1.94	0.32	48
*Odontamblyopus rubicundus*	Taenioididae		2	0.161	0.027	0.053	0.002	0.107	0.028	3.34	0.35	67
*Odontamblyopus lacepedii*	Taenioididae		3	0.077	0.000	0.033	0.009	0.044	0.009	2.39	0.53	57
Mean biomagnification factor	Dolphin liver					350		14.3		6.8		
Mean biomagnification factor	Dolphin kidney					44.3		9.6		3.4		
Mean biomagnification factor	Dolphin muscle					4.78		18.7		0.8		

The highest mean concentration of THg was found in dolphin liver tissue (32.3±59.0 µg g^−1^ dw), whereas a lower concentration was found in the kidneys (4.52±5.53 µg g^−1^ dw), with the smallest residue level shown in the muscle tissue (1.45±1.62 µg g^−1^ dw). The difference in THg accumulation among the three tissues was significant (*p*<0.05) (**Table 1**). Conversely, a significant difference in MeHg accumulation among different tissues was not found. Dolphin muscle tissue contained the highest MeHg concentration (1.02±1.11 µg g^−1^ dw), followed by liver (0.79±0.61 µg g^−1^ dw) and kidney (0.53±0.49 µg g^−1^ dw). Se concentrations displayed a similar profile as THg, with the highest mean value found in the liver tissue (15.2±19.4 µg sg^−1^ dw), followed by kidney (7.57±3.47 µg g^−1^ dw) and muscle tissue (1.89±1.69 µg g^−1^ dw).

**Figure 2:**
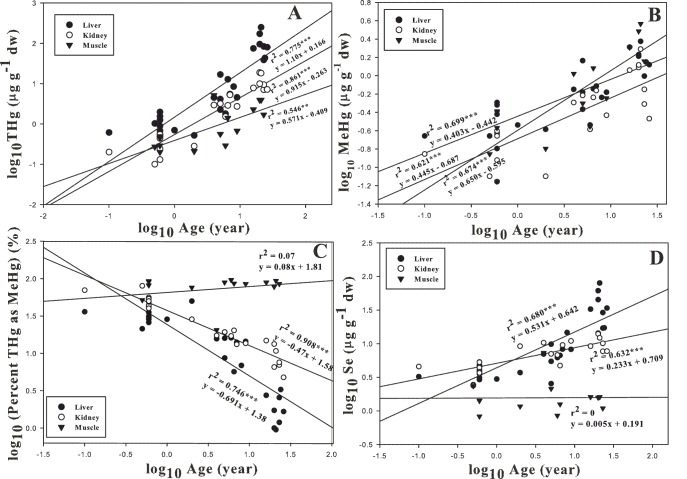
Relationships of dolphin age with the concentrations of THg, MeHg, Se and MeHg/THg ratio in the liver, kidney and muscle tissue, respectively, in the Indo-Pacific humpback dolphins stranded in the Pearl River Estuary (PRE) region.

The log_10_-transformed concentrations of both THg and MeHg in the liver, kidney and muscle tissue of dolphins showed a significantly positive correlation with the log_10_-transformed values of age (**Fig. 2A, B**). However, the slopes between tissue MeHg with age were much lower than those between THg and age for the three tissues. The MeHg/THg ratio in the livers and kidneys significantly decreased with age, while the MeHg/THg ratio in the muscles showed no trend with age (**Fig. 2C**). The mean percentage of MeHg/THg in the liver, kidney and muscle tissue was 18±15, 29±22 and 77±23, respectively (**Table 1**), indicating that the MeHg concentrations in the liver and kidney tissue represented 30% or less of the THg, which was generally lower than the MeHg/THg fraction that appeared in the prey fish (ranging from 18% to 83%, with an average of 42%, **Table 2**). The dolphin muscle tissue showed the highest MeHg/THg ratio. Se accumulation in the liver and kidney tissue significantly increased with age, whereas the concentration of Se in muscle tissue was not obviously affected by age (**Fig. 2D**).

**Figure 3:**
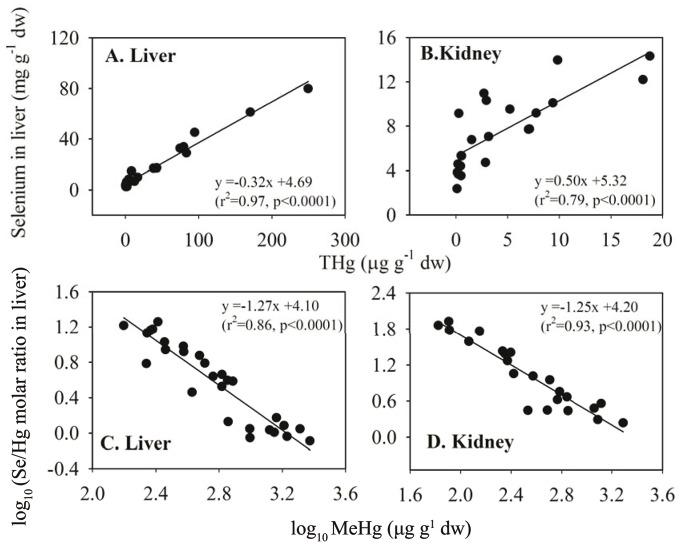
Regression analysis of Se with THg, and of log_10_ (Se/Hg) with log_10_ MeHg in the liver and kidney of Indo-Pacific humpback dolphins (n = 28) stranded in the Pearl River Estuary (PRE) region.

**Figure 4:**
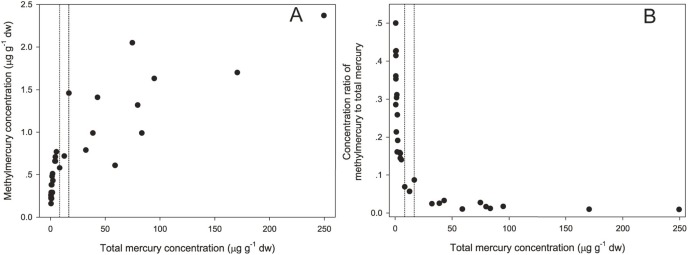
MeHg concentration (µg g^−1^ dw) (A) and concentration ratio of MeHg to THg (B) against THg concentration (µg g^−1^ dw) in the liver samples of the stranded Indo-Pacific humpback dolphins in the Pearl River Estuary region (n = 28).

The mean molar ratio of Se to THg was highest in the kidney tissue (21.5±25.3) and the lowest in the liver (6.04±5.40), with a medium ratio observed in the muscle tissue (6.75±6.26) (**Table 1**). The molar ratios of Se/THg in the livers and kidneys of the juveniles were eight-fold higher than in the adult livers and 14-fold higher than in the adult kidneys, respectively. A significant positive correlation was found between the concentrations of Se and THg in both the livers and kidneys (**Fig. 3**A, B). The log_10_ Se/THg molar ratios showed strongly negative regressions with the log_10_ MeHg levels in the livers (*p*<0.05) and kidneys (*p*<0.05) (**Fig. 3**C, D). A significantly positive relationship between THg and MeHg was observed in the livers, with an inflection range of 8.4–16.9 µg g^−1^ dw of THg (**Fig. 4**).

No significant differences were found in the THg and MeHg concentrations in the liver and muscle tissues between males and females. Only the mean Se concentrations in the liver tissue were significantly different between males and females (*p*<0.05) (**Table 1**).

## Discussion

Compared with the kidneys and muscles, extremely high concentrations of THg were found in the livers of *S. chinensis* (32.3±59.0 µg g^−1^ dw), although the upper limits of the THg concentrations in the livers were lower than those found in dolphins from the waters of Hong Kong (906 µg g^−1^ dw) [27]. This phenomenon may be related to the detoxification function of marine mammal livers in terms of storage and biotransformation. The tolerance limit of THg in mammalian hepatic tissues appears to be within the range of 100 to 400 µg g^−1^ wet weight (ww) [28]. The THg concentrations shown in **Table 1** are below this range; however, a dolphin analyzed in our laboratory was found with a liver THg concentration of 1374 µg g^−1^ dw. Albeit not as high as the results from Japan (1600 µg g^−1^ dw) [29] and the Mediterranean (13150 µg g^−1^ dw) [30], the THg concentrations detected in the liver samples of *S. chinensis* in the PRE were high enough to cause damage to the internal organs of the contaminated individuals.

The accumulation of MeHg tended to increase with age (**Fig. 2**) but at a lower rate than that of THg. No obvious trend existed between the MeHg/THg ratio (%) and age in the muscle samples, whereas the liver and kidney samples showed a decreasing trend of MeHg with age, which could be ascribed to a slow demethylation process that occurs in livers and kidneys but not in muscles [29], [31]. Among the three tissue types, muscle tissue accumulated the highest MeHg concentrations, which is consistent with the hypothesis that lack of demethylation mechanisms occur in muscles [32]. Based on studies of several small mammals [33], the lethal level of MeHg in brain tissue was proposed to be in the range of 12–30 µg g^−1^ ww (equivalent to 60–150 µg g^−1^ dw). However, the highest MeHg concentration in the brain tissue (0.41 µg g^−1^) from our previous study (unpublished data) was significantly lower than the lethal level.

The relationships between age and THg concentration in livers and kidneys of different species of marine mammal have been extensively examined [12], [30], [34], and indicate that the hepatic and renal THg concentrations increase with age. This correlation implies a higher capability for the bioaccumulation of toxic elements than for their elimination throughout these animals' life span. In addition, as the size of the prey and the quantities of food tends to increase in proportion with the growth of the dolphins, the trophic transfer of the toxic metals may also progressively increase [35]. In the present study, the THg concentration increased with age in the livers, kidneys and muscles (**Fig. 2**).

Because of the difficulties in tracking down the prey fish species of dolphins in natural habitats to analyze their tissue contaminant residues, the trophic transfer of THg, MeHg and Se from prey fish to dolphin has scarcely been studied, even though dolphins are considered one of the top predators in the ocean. Through a comparison of the mean concentrations of THg, MeHg and Se from the prey fish to dolphin tissues, our studies outline the first direct evidence of the biomagnification processes of contaminants in this cetacean (**Table 2**). For Hg_inorg_, the average concentrations in the three dolphin tissues were 4.78- to 350-fold greater than the concentrations detected in their prey fish, suggesting that a significant biomagnification process could occur for Hg, especially in the liver. The present study also showed that concentrations of MeHg in the cetacean tissue were 9- to 18-fold higher than the concentrations in the prey fish because of the high assimilation and bioaccumulation capability of MeHg across trophic levels. Conversely, trophic transfer of Se was weak except in dolphin livers. The concentrations of Se in the dolphin muscle tissue were even lower than the concentrations found in the prey fish (BMF<1), which is likely due to the lack of accumulation or rapid elimination of Se in this tissue. The comparison of THg, Se and MeHg between the dolphin organ tissues and prey fish indicated that THg and MeHg could significantly accumulate in dolphin organs. However, whether THg and MeHg can threaten the health of this cetacean is best determined by the molar ratio of Se/THg, which will be discussed below.

In the marine environment, the dominant portion of Hg in fish and squid is present in the form of MeHg [36]. However, the majority of Hg accumulated in the internal organs of cetaceans appears as inorganic Hg(II), especially in the liver, indicating that a demethylation of MeHg occurs in the liver [34], [37]. The formation of the compound Se–Hg, resulting from the combination of a demethylation product (i.e., Hg(II)) and Se, appears to be the last step in the demethylation processes, leading to the fossilization of THg and Se in the form of an inert compound [17], [19], [38]. In the present study, the relationship between the log_10_ Se/THg value and log_10_ MeHg concentration in the livers and kidneys of Indo-Pacific humpback dolphins showed a significant negative correlation (**Fig. 3**). This finding strongly suggests that the Se–Hg compound was formed, causing the fraction of Se/THg in the form of the Se–Hg compound to decrease with respect to the increased MeHg levels [39]. The significant positive relationship between Se and THg and the significant negative correlation between the proportion of MeHg in THg and the THg concentration further confirms that detoxification processes act on MeHg in the livers and kidneys of the dolphins (**Fig. 3** and **Fig. S1**), similar to the results reported for other cetaceans [18], [37].

The molar ratio of Se/THg can be considered an indicator of the extent of toxicity caused by Hg contamination because the toxic effects are alleviated after the formation of the Se–Hg compound, which is stable and has no relevant biological impact. Se can be combined with other elements to form various compounds. A molar ratio between Se and THg higher than 1 implies that Se is providing potential protection against Hg toxicity. However, a ratio below 1 suggests limited Se protection against Hg toxicity [40], [41]. In the present study, the average molar ratios of Se to THg in the liver, kidney and muscle tissue were 6.03, 21.48 and 6.75, respectively, which are all significantly higher than 1. The high Se/THg ratios in tissues were due to the fact that juveniles comprised most of the analyzed samples and low THg concentrations were found in these samples. However, certain adults had molar ratios below 1 and were in danger of suffering Hg toxicity. According to Caurant et al. [38], young animals were still unable to demethylate MeHg efficiently, leading to higher mean percentages of MeHg/THg concentrations in juveniles than adults. We concluded that a molar ratio of Se/THg of 1 in the organs of adults can be used as an indicator of demethylation processes in the organs. The mean molar ratios of Se/THg in the liver samples of adult dolphins were approximately 1, whereas the mean value in the kidneys was 2.65. However, this pattern does not preclude the possibility of the demethylation process occurring in the kidneys. Se, as the constituent of selenoprotein P and selenoenzymes, also plays an important role in maintaining the normal functions of the kidneys in addition to its detoxification function against Hg.

The relationship between THg and MeHg in dolphin livers may indirectly reveal how demethylation occurs. In this study (**Fig. 4**A), when the THg concentration was lower than 8.4 µg g^−1^ dw, the MeHg concentration increased significantly, indicating that no demethylation occurred when the body burden of THg was low. However, when the THg concentration increased from 8.4 µg g^−1^ dw to 16.9 µg g^−1^ dw, the increase of MeHg in the livers slowed down because the demethylation process was presumably occurring, although we lack the direct chemical and biochemical evidence to confirm this assumption. The concentration ratio of MeHg to THg was 1.86± 0.8% (Mean ± SD, n = 9) when the THg concentration exceeded 16.9 µg g^−1^ dw, whereas the concentration ratio was 27±11% (Mean ± SD, n = 16) when the THg concentration was below 8.4 µg g^−1^ dw (**Fig. 4**B). Therefore, it is assumed that the demethylation processes could be activated when the THg concentration reaches a threshold range (i.e. 8.4–16.9 µg g^−1^ dw). According to Palmisano et al. [42], mercury is generally stored as MeHg in the livers of dolphins initially, and after a threshold value (100 µg g^−1^ ww) is reached, demethylation takes place with the co-accumulation of Se/THg at a molar ratio of 1∶1. In the livers of *S. chinensis*, Se was significantly related to THg (R^2^ = 0.9740, *p*<0.05), and the molar ratios of Se/THg in most our samples were far higher than 1 (**Fig. S2**), suggesting that Se was involved in the detoxification process of MeHg.

The co-accumulation of Se/THg with a molar ratio of 1∶1 in the livers of cetaceans after a threshold value is reached has been reported by several authors in striped dolphins and Dall’s porpoises [42], [43]. Together, these results indicated that exceeding the threshold value of THg was a necessarily initial step before triggering the demethylation processes. Compared with the threshold value of striped dolphins (100 µg g^−1^ ww) and Dall’s porpoises (20–30 µg g^−1^ dw), a lower THg concentration was found in the present study for *S. chinensis*. These differences could be attributed to the metabolism and varying foraging habits among the cetacean species, which might also influence the accumulation of Hg and Se, as reflected in the Se/THg molar ratios [12], [44]. The lower threshold of hepatic THg concentrations for the equimolar accumulation of Se and Hg in *S. chinensis* suggests that this species has a greater sensitivity to THg concentrations than is found in striped dolphins and Dall’s porpoises.

## Conclusions

This study is the first comprehensive investigation on the distribution and fate of THg, MeHg and Se in livers, kidneys and muscles of Indo-Pacific Humpback Dolphins collected for almost 10 years from the PRE of China, and in the prey fishes from the PRE. The results clearly showed that THg and Se were mainly accumulated in dolphin livers while the highest residue of MeHg was found in dolphin muscles. This study contains the first experimental evidence for the potential trophic transfer from the fish to Indo-Pacific Humpback dolphins, showing a high biomagnification factor of Hg_inorg_ in liver (350×) and MeHg in muscle (18.7×). Our studies suggest that the Indo-Pacific humpback dolphins may have the potential to detoxify Hg via the demethylation of MeHg and the formation of tiemannite (HgSe) in the liver and kidney.

## Supporting Information

Figure S1 **Relationship between log THg (µg g^−1^ dw) and percentage of MeHg/THg in the liver, kidney and muscle of** ***Sousa chinesis*** **stranded in the PRE.**
(TIF)

Figure S2 **(A) Individuals with molar ratio of Se/Hg higher than the threshold range (8.4–16.9 µg g^−1^ dw). (B) Individuals with molar ratio of Se/Hg lower than the threshold range (8.4–16.9 µg g^−1^ dw).**
(TIF)

Table S1 Sampling information of *Sousa chinensis* stranded in the Pearl River Estuary.
(DOCX)
