# Supplementary figures and images for: Mercury and Selenium in Stranded Indo-Pacific Humpback Dolphins and Implications for Their Trophic Transfer in Food Chains
Source: PLoS One. 2014 Oct 13;9(10):e110336. doi: 10.1371/journal.pone.0110336 (PMC4195725; doi:10.1371/journal.pone.0110336)

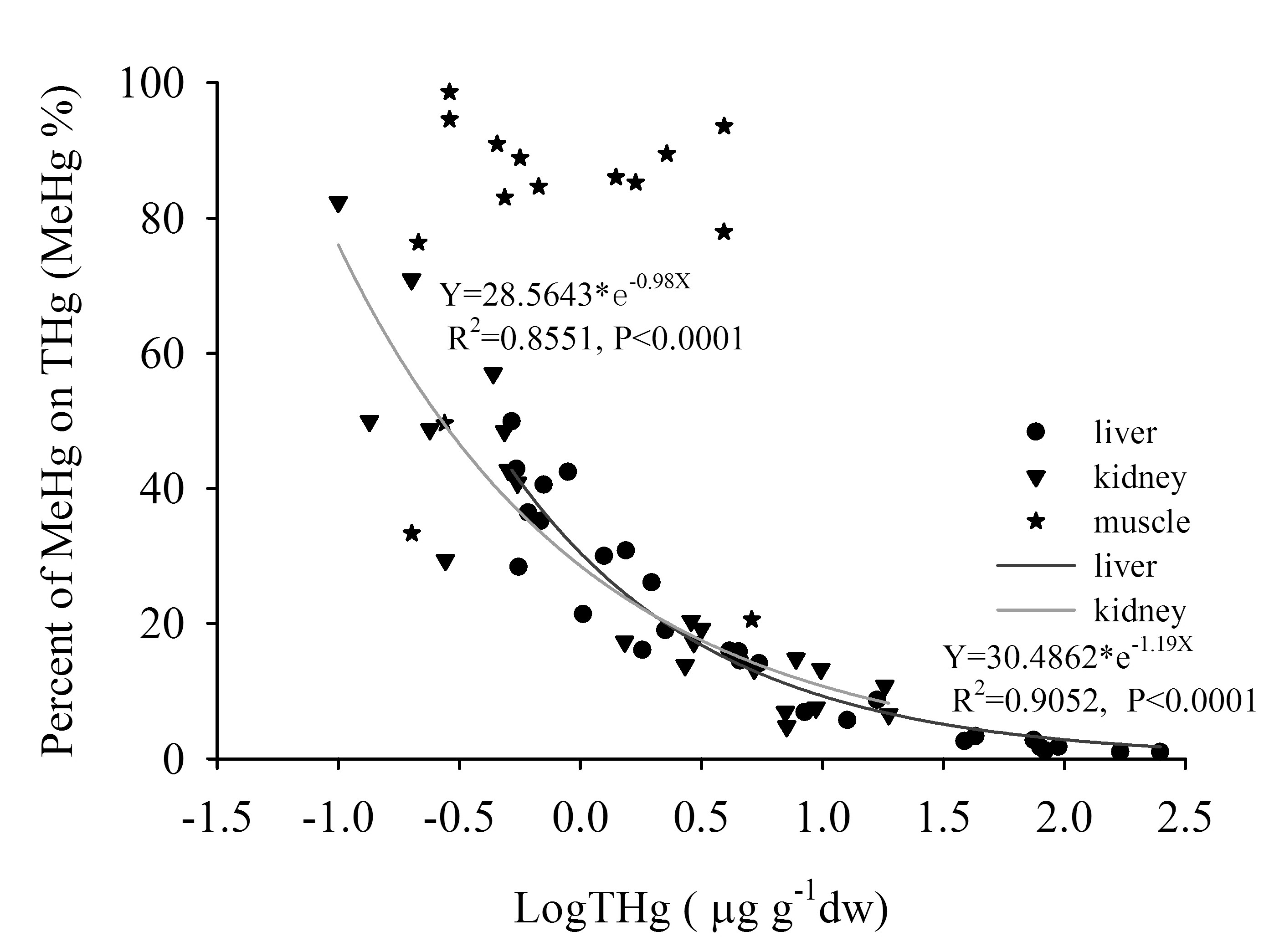

Supplement: Figure S1 — Relationship between log THg (µg g−1 dw) and percentage of MeHg/THg in the liver, kidney and muscle of Sousa chinesis stranded in the PRE. (TIF) [file pone.0110336.s001.tif]
