# Supplementary material for: Mercury and Selenium in Stranded Indo-Pacific Humpback Dolphins and Implications for Their Trophic Transfer in Food Chains
Source: PLoS One. 2014 Oct 13;9(10):e110336. doi: 10.1371/journal.pone.0110336 (PMC4195725; doi:10.1371/journal.pone.0110336)
Supplement: Table S1 — Sampling information of Sousa chinensis stranded in the Pearl River Estuary. (DOCX) [file pone.0110336.s003.docx]

| ID Number | Date of collection | Site | Sex | Length | Age |
| --- | --- | --- | --- | --- | --- |
| 1 | 20100425 | Qi’ao Island, ZH | M | 79.5 | <1 |
| 2 | 20120104 | Xiangzhou Port, ZH | F | 106 | <1 |
| 3 | 20091028 | Gongbei, ZH | M | 107 | <1 |
| 4 | 20100722 | Beauty Bay, ZH | M | 109 | <1 |
| 5 | 20110102 | Yeli island, ZH | F | 113 | <1 |
| 6 | 20101201 | Beauty Bay, ZH | M | 113.5 | <1 |
| 7 | 20040806 | Nanshui Town, ZH | F | 115 | <1 |
| 8 | 20100716 | Gongbei, ZH | M | 118 | <1 |
| 9 | 20090208 | Gongbei, ZH | M | 162 | 0.5 |
| 10 | 20041111 | Jiuzhou Port, ZH | F | 179 | 1 |
| 11 | 20070402 | Beauty Bay, ZH | M | 190 | 9 |
| 12 | 20100306 | Qi’ao Island, ZH | M | 191 | 2 |
| 13 | 20091029 | Xiangzhou Port, ZH | M | 203 | 5 |
| 14 | 20050124 | Guishan Island, ZH | M | 208 | 5 |
| 15 | 20100322 | Hebao Island, ZH | F | 215 | 4 |
| 16 | 20110915 | Qiye Ling, ZH | F | 222 | 6 |
| 17 | 20100722 | Tangjia Town, ZH | M | 223 | 7 |
| 18 | 20101003 | Gongbei, ZH | M | 225 | 6.5 |
| 19 | 20070404 | Dieshi Beach ,ZH | F | 230 | 5 |
| 20 | 20090419 | Couple Road, ZH | M | 233 | 8 |
| 21 | 20090323 | Niupo Bay, ZH | M | 236 | 23 |
| 22 | 20080316 | Jiuzhou Port, ZH | F | 237 | 24 |
| 23 | 20080819 | Beauty Bay, ZH | M | 241 | 16 |
| 24 | 20080614 | Nanhai, FO | F | 251 | 20 |
| 25 | 20100306 | Qiye Ling, ZH | F | 254 | 23 |
| 26 | 20110505 | Nanhai, FO | F | 254 | 25+ |
| 27 | 20070704 | Jiuzhou Port, ZH | M | 268 | 20 |
| 28 | 20070920 | Guishan Island, ZH | F | 269 | 21 |

**Table S1** Sampling information of *Sousa chinensis* stranded in the Pearl River Estuary.
